# Charge Transport Regimes of MoS_2_ Nanosheets at Cryogenic Temperatures: Implications for Cryogenic Electronics

**DOI:** 10.1021/acsanm.5c04844

**Published:** 2025-11-27

**Authors:** Michael D. Thompson, Matthew Haworth, Owain T. Hughes, Jonathan Prance, Yuri Pashkin, Luca Panarella, Farzan Gity, Gioele Mirabelli, Giorgos Fagas, Ray Duffy

**Affiliations:** † Department of Physics, 4396 Lancaster University, Lancaster LA1 4YB, U.K.; ‡ Tyndall National Institute, 261183 University College Cork, Lee Maltings, Cork T12 R5CP, Ireland

**Keywords:** field-effect transistor, MoS_2_, charge transport, variable range hopping, thermal activation

## Abstract

The electron transport of n-type back-gated MoS_2_ field-effect transistors is investigated in the temperature range of 0.3–271 K. The electrical characteristics exhibit significant variations in the drain current in the subthreshold region, when the drain voltage is sufficiently low. The data analysis reveals two distinct charge transport mechanisms at high temperatures. At high gate voltages, charge transport is well described by variable range hopping theory, which suggests the Efros–Shklovskii regime, while at low gate voltages, we observe a transition to the conventional thermal activation regime. The observed phenomena are at considerably greater device dimensions compared to previously reported, as going to sub-Kelvin regimes loosens the dimensional restraints on the device. Moreover, a huge temperature-dependent threshold voltage shift (δ*V*_TH_/δ*T*) is observed in the whole temperature range, approximately 110 mV/K, incredibly spanning as much as 30 V, with *V*_TH_ increasingly more positive with decreasing temperature. Evidence of a resistive network for charge carriers is also seen, as there appeared to be parallel channels of conduction within the FETs, each with a different threshold voltage. All this physics needs to be factored in should 2D material FETs be considered for quantum electronics at cryogenic temperatures.

## Graphical abstract

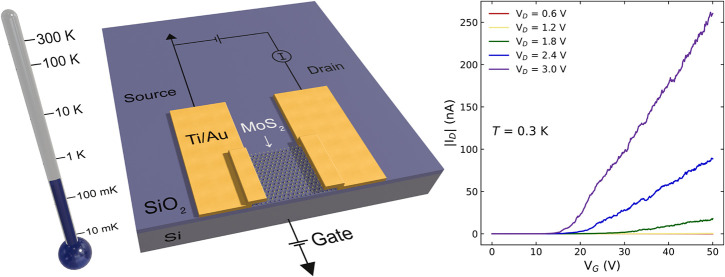

## 1 Introduction

Quantum nanoelectronics is concerned with charge transport in nanoscale devices functional in the quantum regime. Electronic studies at sub-Kelvin temperatures are necessary to understand both a material’s suitability in quantum devices and the physics of the device characteristics. For example, group IV and III–V nanostructured devices have been typically characterized by electron transport measurements biased across cryogenic temperatures (mK-K) and at low-to-high frequencies (DC-GHz) to be used for charge- or spin-based qubit formation, manipulation, and operation. In this work, we report on functional MoS_2_ field-effect transistor (FET) devices down to the sub-K regime and analyze their transport characteristics in order to understand the capabilities of, and physics in, transition metal dichalcogenides (TMDs) applied to quantum nanoelectronics.

Two-dimensional (2D) TMD materials not only present themselves as promising candidates for advanced transistor scaling and ultrahigh integration of solid-state electronic switching devices but also offer a wide range of material properties and the possibility of forming artificial quantum objects, such as single-photon emitters, quantum dots, and topological states, paving the way for their application in quantum information science.^1^ For analog devices, 2D semiconductors are also particularly suitable for building substrate-agnostic integrated circuits, such as operational amplifiers,^2^ which could, for example, be directly integrated with superconducting quantum circuits at cryogenic temperatures.

Should functional TMD FETs be fabricated for quantum or space applications, one must be aware of new physical phenomena or peculiarities that become evident once thermal noise is reduced. To date, the few studies dealing with TMD-based FETs at cryogenic temperatures reported working devices at temperatures no lower than a few Kelvin degrees. These studies are also mainly focused on evaluating performance^3^ or obtaining low-resistance ohmic contacts,^4^ rather than investigating the underlying physics of 2D materials’ FET electronic behavior at very low temperatures. Here, we demonstrate sub-K operation of n-type back-gated MoS_2_ FETs and study their electrical characteristics in these operating regimes, up to room temperature, aiming at characterizing the new features in the charge transport of the MoS_2_ material.

Overall, apart from a few select works, such as that by Hamilton,^5^ with differential conductance maps at <10 mK, the electrical properties of 2D TMDs have not been adequately examined at the sub-K threshold. Kim et al. investigated how the electrical properties of Ohmic van der Waals contacts of MoS_2_/In interfaces such as conductance, carrier mobility, and contact resistance vary with temperature in cryogenic regimes.^6^ They observed Ohmic-like transport based on a field-emission mechanism in the contacts from 2.4 to 300 K, in addition to a small interfacial charge. Similarly, Gao et al.^7^ examined the transfer characteristics, transconductance, and radio frequency performance of chemical vapor deposition-grown MoS_2_ films, demonstrating maximum extrinsic and intrinsic cutoff frequencies of 16 and 24 GHz as well as a maximum transconductance of 35 μS/μm at 4.3 K. Optimal electrical performance of alloy contacts with other 2D TMDs at comparable temperatures was demonstrated in WS_2_, examining carrier mobility versus temperature, at different drain voltages, as well as contact resistance versus temperature, down to 3 K.^8^ Notably, the devices exhibited low contact resistances of ≈10 kΩ·μm at 3 K and Schottky barrier heights of 1.7 meV. The electrical characteristics of other electronic devices such as memtransistors, which have potential applications in neuromorphic computing, have also been extensively studied by Sangwan et al.^9^ Subtleties, such as hysteresis differences in the transfer characteristics, between FETs and memtransistors should be considered when comparing these different devices.

## 2 Experimental Methods

### 2.1 Sample Fabrication

In this work, MoS_2_ devices were fabricated in the standard way using exfoliated flakes from a single crystal;^10–12^ further information can be found in the Supporting Information and Figure S1. In short, devices were fabricated by mechanical exfoliation from MoS_2_ single crystals, followed by the transfer of the resulting layers onto Si/SiO_2_ substrates, and completed with Ti/Au metal contact pad formation as shown in Figure 1a.

**Figure 1:**
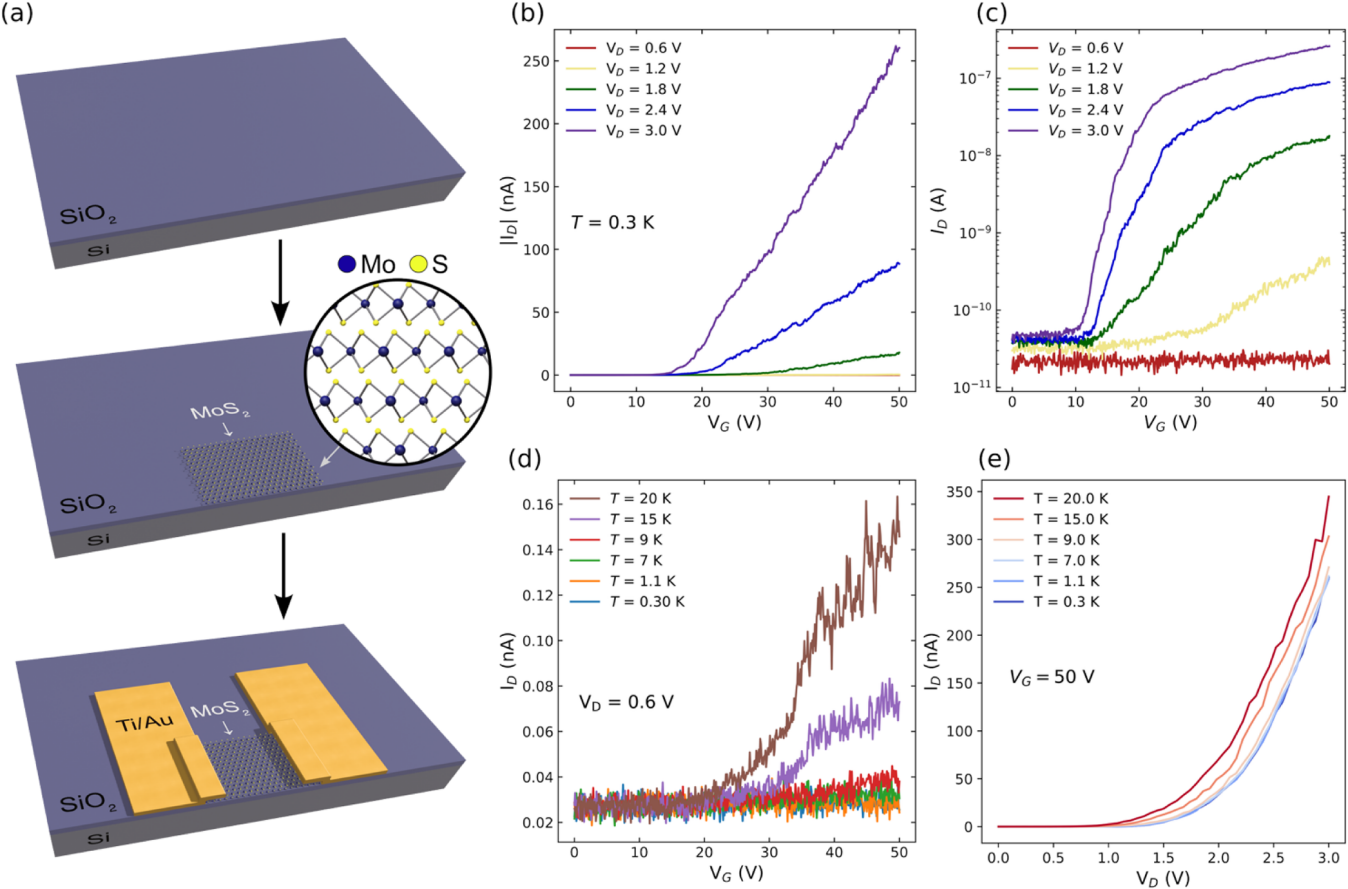
(a) Schematic of the device fabrication. A few nm thick flake of exfoliated MoS_2_ is placed onto a SiO_2_/Si substrate followed by deposition of Ti/Au contacts. (b) and (c) Representative transfer characteristics of an n-type MoS_2_-based MOSFET at 0.3 K, with varying drain potential and (d) at low *V*_D_ (0.6 V), with temperature varying from 0.3 to 20 K. (e) Current–voltage characteristics for *V*_G_ = 50 V at varying temperatures.

### 2.2 Electrical Characterization

Prior to cryogenic testing, devices were initially checked at room temperature to verify basic FET operation, such as current conduction and gate-bias current modulation over several orders of magnitude. Cryogenic measurements were taken in an Oxford Instruments Triton 400 dilution refrigerator (<1 K) and an Oxford Instruments IO 1 K refrigerator (1–300 K) with a 2-terminal configuration using a Keithley 2400 source-measure unit (SMU) and a second SMU for applying a back-gate voltage.

## 3 Results and Discussion

Figure 1b,c shows representative transfer characteristics of a MoS_2_-based FET at 0.3 K. It is greatly encouraging to observe functional transistor operation of MoS_2_ devices at sub-Kelvin temperatures, making this family of devices viable to integrate in quantum electronic circuits that operate at these temperatures. For clarity, the same data are plotted with linear and log *y*-axis scales in Figure 1b,c. The device is turned on at positive gate potentials (*V*_G_), with a drain current (*I*_D_) that increases with increasing positive gate voltage, suggesting an n-type behavior. The drain current does not saturate within the investigated potential range, as seen in the current–voltage (IV) characteristics in Figure 1e which also shows the diode-like effect of the IV curves at low temperature. Further IV characteristics are shown in Figures S4 and S5 where we observe the IV to be linear at room temperature with only a weak tendency toward saturation observed at low gate voltage. A clear shift of the threshold voltage (*V*_TH_) is observed caused by varying the drain potential (*V*_D_), possibly due to drain-induced barrier lowering. Note the significant current variations of the *I*_D_ versus *V*_G_ characteristics present in the different curves visible in Figure 1b,c. Such variations cannot be ascribed to instrumental noise due to very low signals, as they are significantly above the sensitivity limit of the measurement setup. Notably, Lee et al. observed current variations in monolayer MoS_2_ at 6 K with *V*_D_ = 100 μV, ascribed to the Coulomb blockade effect.^13^ Equivalent behavior was seen in a p-type MoS_2_-based FET, shown in Figure S2, where that device was fabricated from a flake exfoliated from an intentionally Nb-doped MoS_2_ crystal, and the flake in question was 9 nm thick. In the remainder of this article, we will focus on the n-type MoS_2_-based FETs for consistency.

The above-mentioned variations are more clearly presented in Figure 1d. Note that the same data are plotted with a log-scale *y*-axis in Figure S3. Here, the transfer characteristics at *T* = 0.3–20 K, *V*_D_ = 0.6 V, and *V*_G_ = 0–50 V are shown. This unusual characteristic was reproducible across many low temperatures and appeared in both n- and p-type FETs, particularly noticeable at low *V*_D_ and in the subthreshold region. In most circumstances, when studying large-dimension metal-oxide-semiconductor field-effect-transistor (MOSFET) device behavior, say Si MOSFETs at room temperature, one would not expect to see such variations in current.

For comparison, Si MOSFETs have been studied at cryogenic temperatures,^14–22^ with many works focusing on the subthreshold slope during turn-on. In some cases, small-area Si MOSFETs exhibit variations in subthreshold current at deep cryogenic temperatures. Such variations have been ascribed to the density of states band-tails, or carriers undergoing resonant tunneling through the channel, or percolation theory, due to electrostatic potential fluctuations caused by interface charges.^23,24^ Finally, very recently Cherkaoui et al. studied high-k dielectric/InGaAs interface defects at cryogenic temperatures^25^ and showed that the exchange of free carriers between oxide states and the conduction or the valence band is strongly temperature-dependent.

At higher temperatures (>30 K), variable range hopping (VRH) has been identified as the transport mechanism in previous reports on WS_2_ ^8^ and MoS_2_.^26–29^ This form of transport manifests itself as a temperature-dependent resistivity of the following form:

1 $$\rho =\rho_{0}\exp{\left(T_{0}T\right)}^{p}$$

where ρ_0_ = *AT* ^*m*^ (*m* is an empirical prefactor exponent), *T*_0_ is a characteristic temperature, and *p* = 1/(*d* + 1), where *d* is the effective dimensionality of the system.^30^ VRH hopping occurs in compensated semiconductors at temperatures where impurities are frozen out and electrons hop between impurities rather than entering the conduction band.^31^

One signature of VRH transport is a linear dependence of ln­(ρ/*T^m^*) against *T^–p^* as reported previously;^32,33^ however, if *T*_0_ is small, then at high temperature, the exponential in eq 1 becomes sufficiently small that this dependence can be fit linearly for any realistic value of *p*. In order to identify whether such transport is evident in our samples, we apply eq 1 to the current versus temperature data, using *m* = 1, in order to extract *p* for different gate voltages, as seen in Figure 2a, noting that ρ ∝ 1/*I*_D_ as the device is in the linear region. From this, we can determine *p* as a function of *V*_G_ seen in Figure 2b. Here, we find that *p* tends to saturate at a value close to 1/2 at higher gate voltages. While this would suggest that the dimensionality of the electron system is 1, it is more likely a signature of Efros–Shklovskii hopping^33^ where the Coulomb interaction opens up a gap in the density of states.

**Figure 2:**
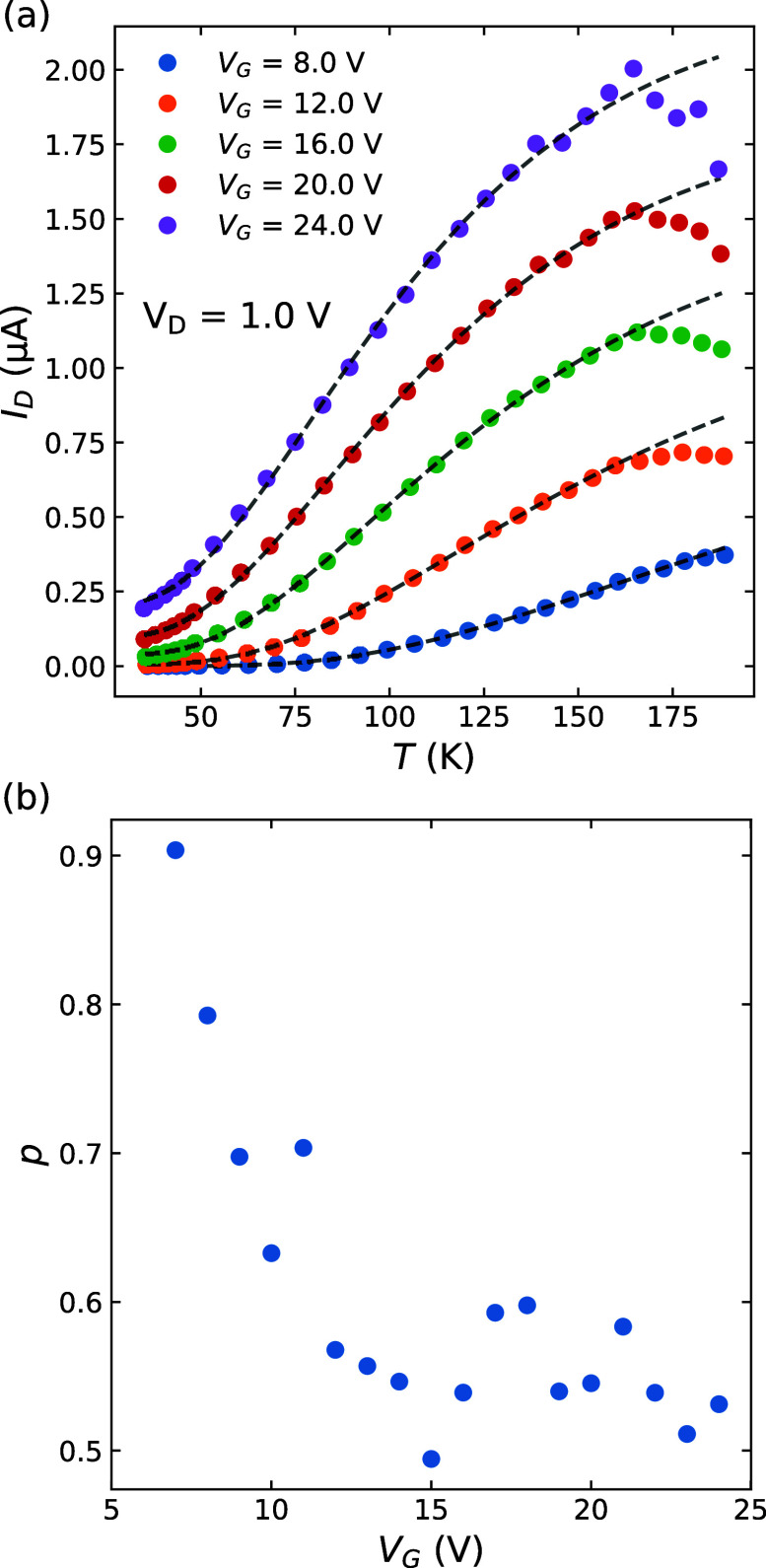
Variable range hopping theory applied to the data (a) fitting eq 1 (dashed lines) for a selection of gate voltages with *V*_D_ = 1.0 V. The fitting starts to deviate above 160 K. The extracted values for *p* across a wider range of gate voltages are shown in (b). Here, we see that *p* tends to saturate slightly above 1/2. See Figures S4 and S5 for additional information about the linear regime of the device at these biases and temperatures.

At lower gate voltages, VRH hopping does not appear to be the dominant transport mechanism. Figure 3 shows an Arrhenius plot of *I*_D_ for low gate voltages indicating that charge transport appears to be thermally activated for *V*_G_ < 8 V,

2 $$I=I_{0}e^{-E_{A}/k_{B}T}$$

where *I*_0_ is a prefactor, *k*_B_ is the Boltzmann constant, and *E*_A_ is the activation energy and is shown as a function of gate voltage in the inset of Figure 3. At *V*_G_ > 8 V, the temperature dependence cannot be explained by thermal activation, presumably due to the transition to the VRH regime. Thermionic emission was also considered, however there was no evidence of this in the data. Above 175 K, we observed a change in the device characteristics for which we currently do not have an explanation and requires future study.

**Figure 3:**
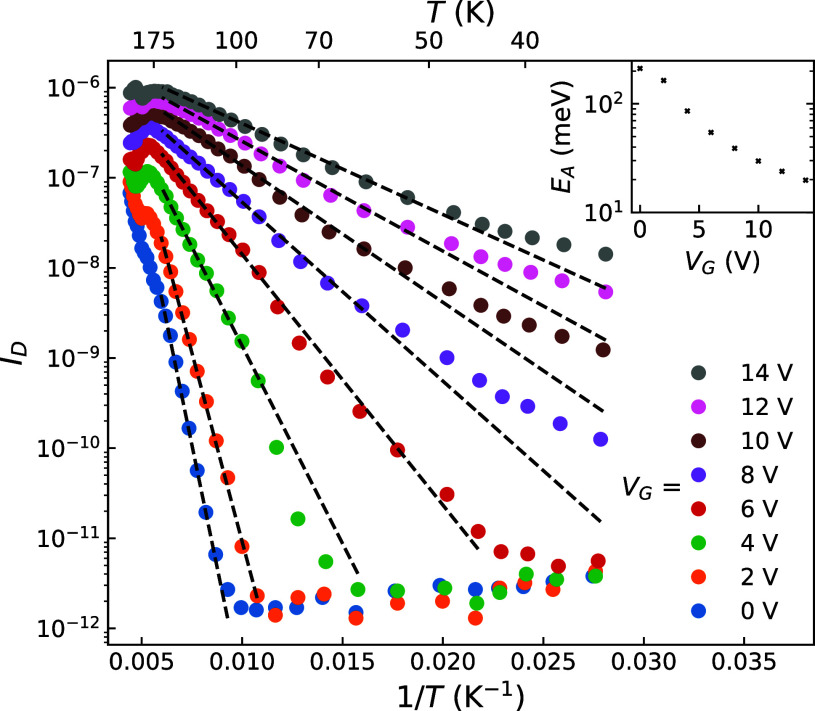
Arrhenius plot of *I*_D_ for different back gate voltages with *V*_D_ = 1.0 V. The dashed lines are linear fits to temperatures 80 K < *T* < 165 K according to eq 2. Fits have been extended beyond the fitting region to emphasize the nonlinearity at lower temperatures. The inset shows the activation energy *E*_A_ as a function of gate voltage.

Figure 4a shows representative *I*_D_ versus *V*_G_ characteristics with *V*_D_ = 1 V across a very wide range of temperatures, increasing from 1.2 to 272 K. N-type transfer behavior is observed as before. However, *V*_TH_ and the on-state current (*I*_D‑ON_) change drastically. Estimating *V*_TH_ by a simple threshold of 1 nA, we find that with lower temperatures, *V*_TH_ increases by approximately 30 V, which could have a significant impact of design for MoS_2_-based circuits, where typically one does not want large shifts in performance as temperature changes. *I*_D‑ON_ decreases by about 4 orders of magnitude, while still appearing to plateau for *V*_G_ ≫ *V*_TH_. This positive shift in *V*_TH_ with lower temperature indicates that it is more difficult to invert a channel or to accumulate charge carriers, while the reduced *I*_D‑ON_ indicates that the channel is more resistive. The trend of increased device resistance with decreasing temperature is consistent with our previous works on 2D material devices.^34,35^ Of course, carrier mobility can drop due to increased impurity scattering at lower temperatures, but a 4 orders of magnitude drop in *I*_D‑ON_ would require an equivalent drop in mobility, as established MOS theory suggests *I*_D‑ON_ and mobility have a linear relationship. There is evidence of δ*V*_TH_/δ*T* in the work of Ghatak et al., in 3- and 1-layer MoS_2_, with their data presented and discussed in terms of conductivity.^32^

**Figure 4:**
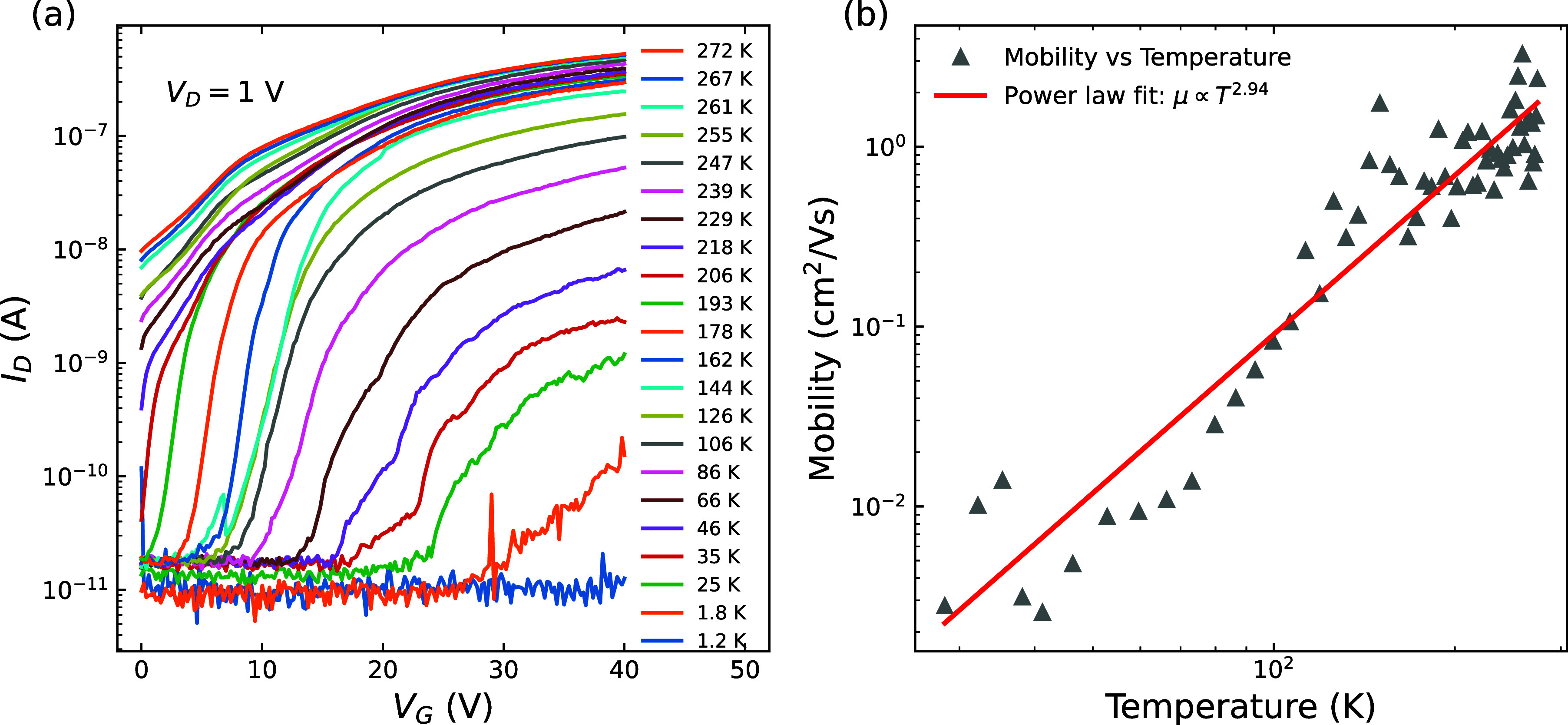
(a) Representative transfer characteristics of an MoS_2_-based MOSFET at 1.2–271.5 K with *V*_D_ = 1 V. An approximate 110 mV/K temperature-dependent VTH is observed spanning approximately 30 V, increasingly more positive with decreasing temperature. (b) Mobility as a function of temperature for the data in (a).

A >100 mV/K temperature-dependent *V*_TH_ is worth contrasting with values found in Si devices, and it is important to consider the underlying cause. In early works, Tzou et al.^36^ and Klaassen et al.^37^ observed a positive *V*_TH_ shift (δ*V*_TH_/δ*T*) in n-type FETs with decreasing temperature, as we do, however, with a range of 1–2 mV/K, vastly lower than what we observe in MoS_2_. Furthermore, d’Alessandro et al.^38^ did a survey of different models for δ*V*_TH_/δ*T*, while Nishida et al.^39^ provided a very thought-provoking discussion and analysis on the potential sources of δ*V*_TH_/δ*T* in terms of the equation:

3 $$V_{\mathrm{TH}}=V_{\mathrm{FB}}+2\phi_{f}+\frac{\sqrt{4q\varepsilon_{0}\varepsilon_{\mathrm{Si}}N_{a}\phi_{f}}}{C_{\mathrm{ox}}}+\frac{eD_{\mathrm{it}}}{C_{\mathrm{ox}}}$$

where *V*_FB_ is flat band voltage, the voltage where no band bending occurs, ϕ_f_ is the difference between the Fermi level (*E*_F_) and the intrinsic level (*E*_i_), *e* is the electron charge, ε_0_ and ε_Si_ are the vacuum and Si relative dielectric constants, *N*_a_ is the doping concentration level, *D*_it_ is the interface state density, and *C*_ox_ is the gate capacitance. Note, this equation is included here to aid the discussion, rather than for fitting or parameter extraction purposes. Thus, δ*V*_TH_/δ*T* is subject to several potential causes, as many of the parameters in eq 3 are temperature-sensitive or contain factors that are temperature-dependent, and this should be studied in more depth in follow-up works to identify the key sources of δ*V*_TH_/δ*T* and reduce this effect in MoS_2_ devices.

Figure 4b shows the mobility as a function of temperature, extracted using the H-function method by Chien et al.^40^ An increasing mobility with temperature is typical when impurity scattering dominates^41^ and a fit to the data shows a T^3^ dependence though T^3/2^ is expected. A high level of impurities is consistent with the sample’s doping level and previous reports of high levels of impurities for exfoliated flakes.^42^ The subthreshold swing is observed to have a stronger temperature dependence down to 150 K (Figure S6) and then saturates below this. A similar behavior is seen in Si MOSFETs^18^ where the saturation is due to the presence of a band-tail. From the mobility temperature dependence, this would suggest that such a band-tail in our samples is due to a high level of impurities.

Figure 5 once again shows FET transfer behavior but is now concentrated on the subthreshold region at more negative *V*_G_. It can be observed in some cases that the device turns on in steps or stages, with alternatingly steep or shallow subthreshold slopes. In other words, the current turn-on appears to occur in a number of parallel channels that become active at different *V*_G_. Take for example, the *T* = 159 K curve, there are turn-on events at *V*_G_ = −12, −8, and +1 V, which could be considered as three separate threshold voltages for this device. Many curves in Figure 5, taken at different temperatures, show this type of behavior, which is unusual in FET devices that would normally have a single turn-on or channel formation event. Recently, for fully depleted SOI devices, Catapano et al. proposed a model based on the concept of resistive networks, linked to percolation transport theory.^43^ When the FET is on, in the presence of an inversion channel, transport can occur in randomly distributed low-resistive regions. Crucially, that group modeled the inversion layer as a network of parallel transistors with different aspect ratios (channel width/channel length) *K* and different *V*_TH_. With a variety of parallel channels of different *K* and *V*_TH_, the result is a subthreshold slope with a mixture of “turn-on” points. It should be noted that in the reported works on Si FETs, this arbitrarily distributed low-resistive region effect was only observed in small-dimension (submicron) devices and not observed in micron-scale channels. In this work, not only do we see this in a non-Si device, but we observe this physics at significantly larger device dimensions, thereby relaxing the dimensional restraints on the fabrication processes and device design required to study this distinctive physics. We speculate that this behavior could be due to top or bottom edge conduction or side edge conduction, as grain boundaries are unlikely for exfoliated flakes.

**Figure 5:**
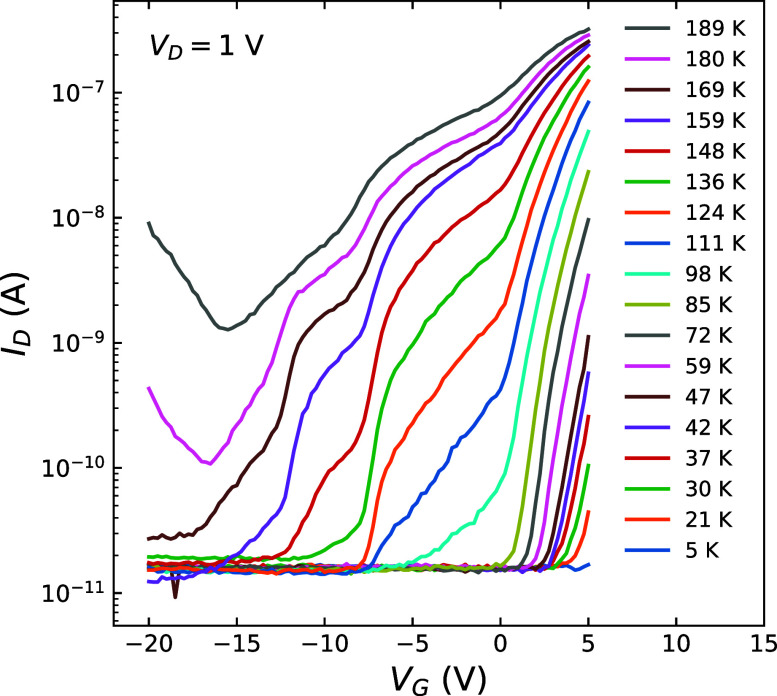
Representative transfer characteristics of an MoS_2_-based MOSFET at 3.7–190.5 K with *V*_D_ = 1 V. These data show a resistor network-type conduction, namely a network of parallel transistors with different aspect ratios, and threshold voltages, *V*_TH_, leading to an unusual subthreshold slope variation. Note also, in comparison with Figure 4, we have measured a number of devices in this work, and when working with exfoliated flakes, there is some unavoidable device-to-device variability; however, the overall trends are the same. This will be solved in future work, namely, when we switch the study to MoS_2_ thin films.

## 4 Conclusions

In conclusion, if TMD FETs are proposed for quantum or space applications, one must be aware of new physical phenomena or peculiarities that become evident once thermal noise is reduced. Based on cryogenic measurements taken in an Oxford Instruments Triton 400 dilution refrigerator down to sub-Kelvin temperatures, MoS_2_ FET devices exhibit a number of distinctive characteristics. Features observed here are at considerably greater device dimensions compared with those previously reported in Si devices. In terms of future directions, a systematic work comparing different metals for contacts on MoS_2_ at sub-K temperatures would be a fascinating study, as there are indications that Sb^44^ and Bi^45^ contacts hold promise. This would enhance our understanding of whether the contact metals are significant in some of these observed phenomena. Conductive atomic force microscopy may help to identify the physical source of the apparent parallel conduction observed. Moreover, as high-frequency behavior and performance are important factors when considering quantum electron devices,^46–49^ future directions for MoS_2_ device characterization at cryogenic temperatures should incorporate these aspects. Finally, one must move toward characterizing devices fabricated from thin-film MoS_2_, rather than flakes, to explore if this physics is still present as we scale up the number of devices in this material system, which is essential in order to develop large-scale integrated circuits. With a large number of devices fabricated from large-area films, we would be able to build a large enough dataset with which we could develop a suitable model to explain the observed behavior, for example, the unusually large temperature coefficient of the threshold voltage.

## Supplementary Material



## Notes

Details of the fabrication process, SEM and AFM images of the devices (Figure S1), transfer characteristics of a p-type MoS_2_-based MOSFET at 0.35 K with the *y*-axis in linear and logarithmic scales (Figure S2), transfer characteristics of an n-type MoS_2_-based MOSFET at low *V*_D_ (0.6 V), with temperature varying from 0.3 to 20 K, with the *y*-axis in logarithmic scale (Figure S3), current–voltage characteristics at 300 K proving the linear regime of the device above *V*_G_ = 0 V (Figure S4), current–voltage characteristics at 34 K showing the “diode”-like effect which is absent at room temperature (Figure S5), and subthreshold swing as a function of temperature (Figure S6) (PDF)
