# Supplementary material for: Charge Transport Regimes of MoS2 Nanosheets at Cryogenic Temperatures: Implications for Cryogenic Electronics
Source: ACS Appl Nano Mater. 2025 Nov 27;8(49):23769–75. doi: 10.1021/acsanm.5c04844 (PMC12706732; doi:10.1021/acsanm.5c04844)
Supplement: Supplementary file 1 [file an5c04844_si_001.pdf]

Supporting Information

Charge Transport Regimes of MoS_2 Nanosheets at Cryogenic Temperatures: Implications for Cryogenic Electronics

Michael D. Thompson*,[1] Matthew Haworth[1], Owain Hughes[1], Jonathan R. Prance[1], Yuri A. Pashkin[1], Luca Panarella,[2] Farzan Gity,[2] Gioele Mirabelli,[2] Giorgos Fagas,[2] Ray Duffy*.[2]

[1] Department of Physics, Lancaster University, Lancaster LA1 4YB, UK.

[2] Tyndall National Institute, University College Cork, Lee Maltings, Cork T12 R5CP, Ireland.

*Corresponding Authors: m.thompson@lancaster.ac.uk, ray.duffy@tyndall.ie

MoS_2 devices were fabricated in the standard way using exfoliated flakes from a single crystal In brief, candidate flakes were identified by a combination of optical microscopy, scanning electron microscopy (SEM), and atomic force microscopy (AFM). Representative images are shown in Fig. S1. For the n-type devices characterized at cryogenic temperatures the flake thicknesses were 55 - 60 nm. Optical lithography was used to pattern Ti/Au contact pads using a standard metal liftoff procedure. The gating of these devices was done via the back of the wafer.

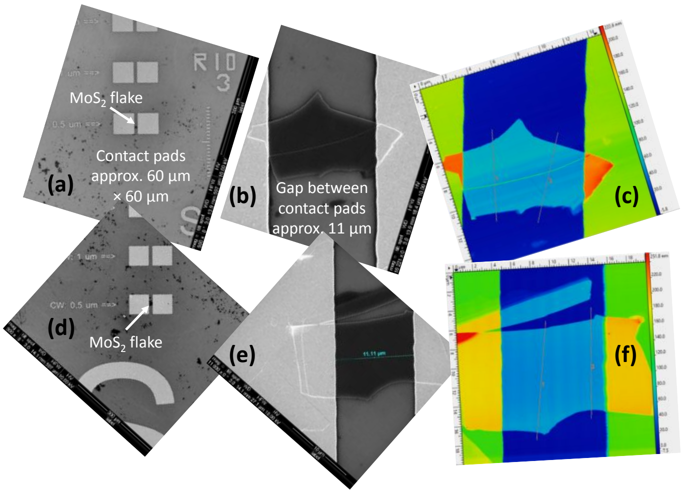

FIG. S1: (a) and (d) Wide view SEM images of the contact pad layout of the device chip. Pads are arranged to connect to an MoS_2 flake underneath. (b) and (e) A closer view shows the overlap of the metal pads with the MoS_2 flake. (c) and (f) AFM was used to determine the flake thickness, (a)-(c) The top row of images shows a MoS_2 flake ("Device A") that is predominantly 55 nm thick, with a kink running across its spine. (d)-(f) The bottom row of images shows a MoS_2 flake ("Device B") that is predominantly 60 nm thick.

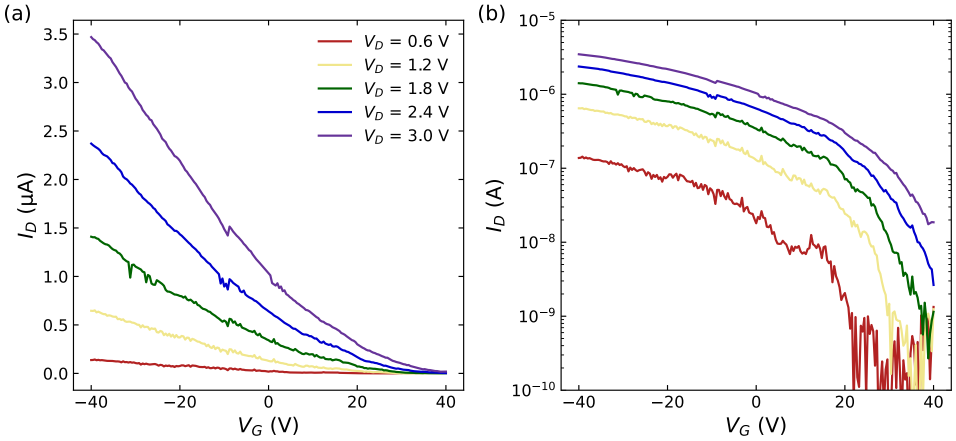

FIG. S2: Representative transfer characteristics of a p-type MoS_2-based MOSFET at 0.35 K, with varying drain potential, with the y-axis in (a) linear scale and (b) logarithmic scale.

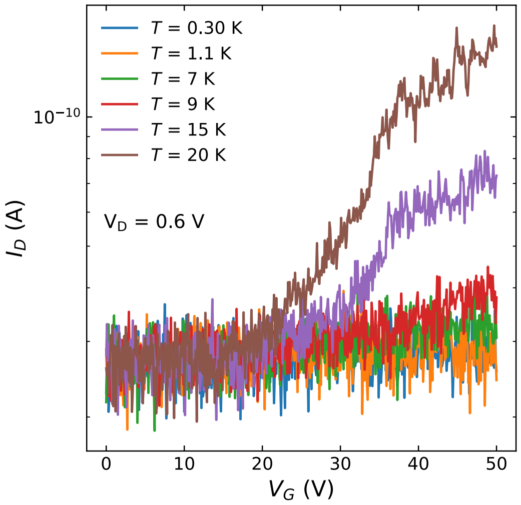

FIG. S3: Representative transfer characteristics of an n-type MoS_2-based MOSFET at low V_D (0.6 V), with temperature varying from 0.3-20 K, with the y-axis in logarithmic scale.

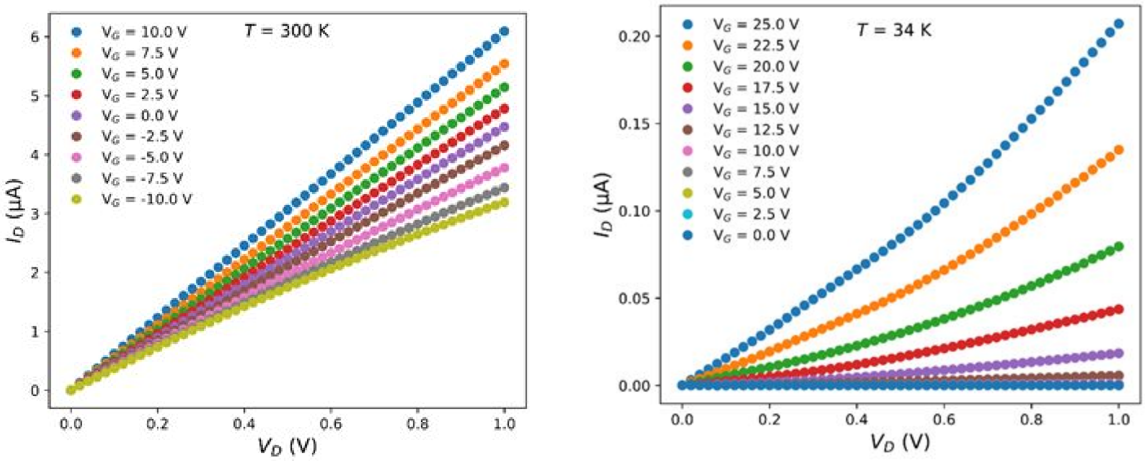

FIG. S4: The 300 K data shows that the device is linear above V_G = 0V. This proves that within the gate voltage range of the analysis, the device is linear.

FIG. S5: Current-voltage characteristics at 34 K. The “diode” like effect is pronounced at low temperature but is absent at room temperature (see FIG. S4).

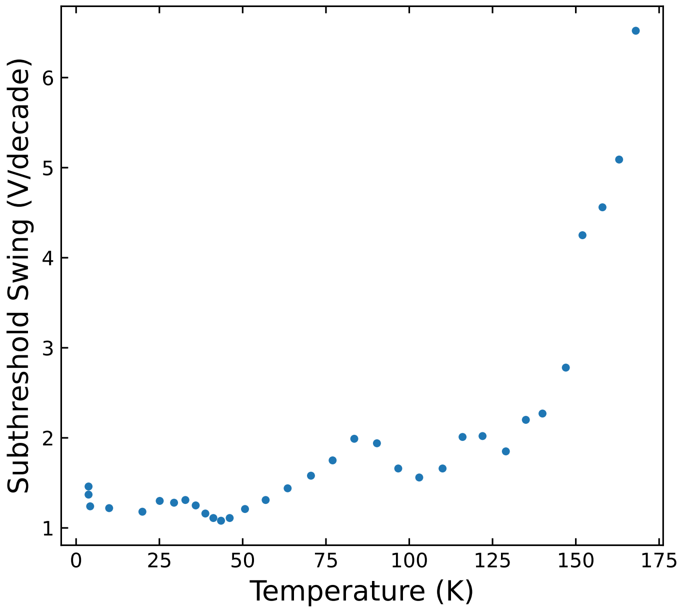

FIG. S6: Subthreshold swing as a function of temperature for the data from Figure 4a which reduces with temperature before beginning to saturate below 150K.

This work was supported by the European Commission through project ASCENT+: Access to European Infrastructure for Nanoelectronics, funded under H2020, (Grant Agreement 871130) and EU H2020 European Microkelvin Platform (Grant Agreement 824109). M.D.T acknowledges financial support from the Royal Academy of Engineering (RF\201819\18\2).
